# HOW ELDERLY PEOPLE INTERACT AFTER THEIR RETIREMENT WITH THE COVID-19 ERA IN JAPAN

**DOI:** 10.1093/geroni/igad104.3052

**Published:** 2023-12-21

**Authors:** Fumihiko Yokota, Mariko Nishikitani, Gretchen Tucker, Dana Bradley

**Affiliations:** Kyushu University, Fukuoka, Fukuoka, Japan; Kyushu University Hospital, Fukuoka, Fukuoka, Japan; University of Maryland, Baltimore County, Baltimore, Maryland, United States; University of Maryland, Baltimore County, Baltimore, Maryland, United States

## Abstract

Amid the COVID-19 pandemic, little is still known about how older Japanese people socialize after retirement. This paper aims to describe the dynamics of social interactions in post-retirement older adults during the COVID-19 pandemic. From January to March 2021, we interviewed 17 Japanese elderly people in Fukuoka and Ehime who were over 65 and have already retired. Face-to-face Interviews were conducted using a semi-structured questionnaire. After the initial transcripts were translated from Japanese to English, we checked the transcripts for accuracy and coded for analysis. The participant’s mean age was 71 years old while their retirement’s mean age was 60 years old. Only one participant had full retirement while the rest were partial retired which they continued to work as part-time workers either with new employers or with the same employers. All participants reported that their social interaction became estranged after retirement, and disrupted during the COVID-19 pandemic. However, participants communicated online and/or interacted face-to-face with (1) neighboring people as a member of community association or volunteers, (2) ex-work colleagues, (3) old schoolmate, (4) their children and/or grand-children, (5) friends with the same hobbies. Following factors that may affect the interactive behavior of Japanese older people were identified; (1) Japan’s healthcare policy and pension system, (2) individual’s socio-economic status, (3) family structure and living arrangement, (4) community’s characteristics, (5) individual’s psychological, cognitive and health status, and (7) individual’s personality. Comparisons of social interactions between Japanese and US older adults will be important to find new knowledge for preventing social isolation.

